# Supplementary material for: Reference set of Mycobacterium tuberculosis clinical strains: A tool for research and product development
Source: PLoS One. 2019 Mar 25;14(3):e0214088. doi: 10.1371/journal.pone.0214088 (PMC6433267; doi:10.1371/journal.pone.0214088)
Supplement: S1 Fig — (DOCX) [file pone.0214088.s003.docx]

| S1 Figure |
| --- |
| 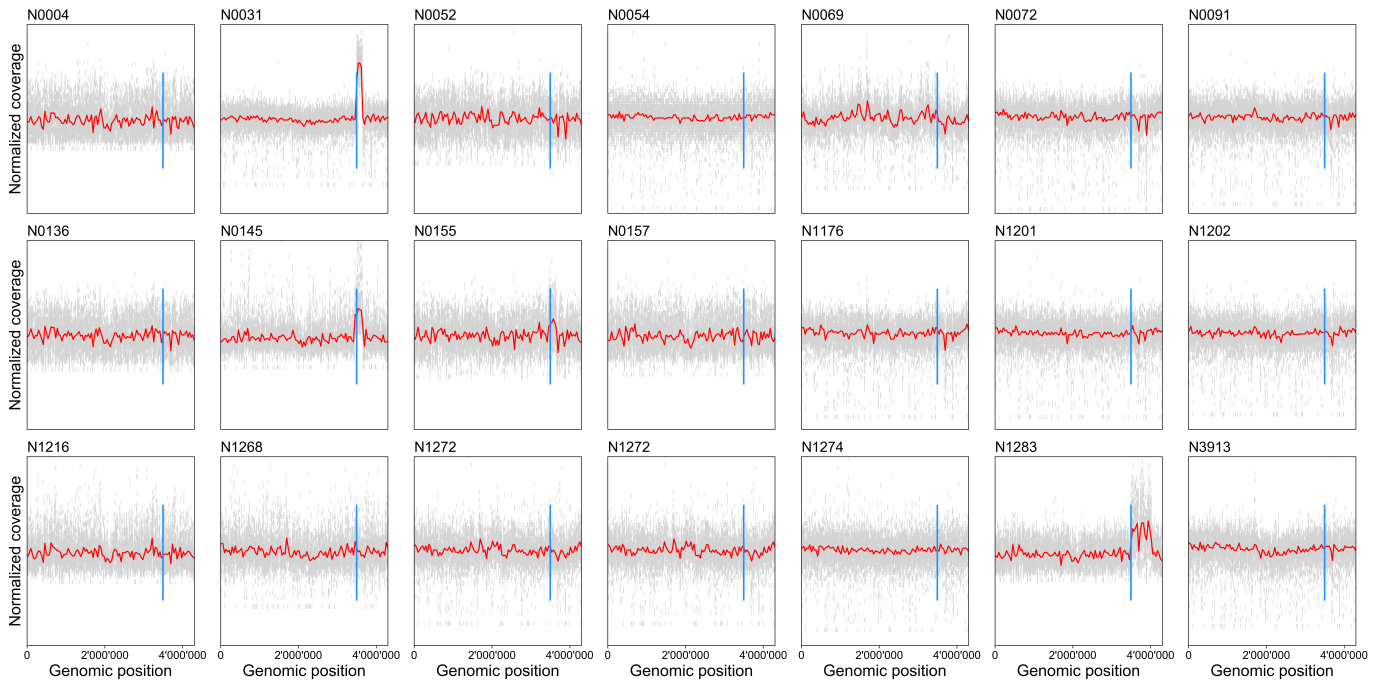 |
|  |

N0031, N0145, N0155 and N1283 carry large genomic duplications. We first calculated the median mapping coverage depth of short sequencing reads for bins of 500 base pairs across the genome and computed their z-score (gray vertical bars). We then calculated the median for 80 such bins spanning 40,000 base pairs (red line). Finally we marked the location of *dosR/dosS* (Rv3133c/Rv3134c, blue bar) to confirm that the duplicated region includes their reading frame.
