# Supplementary material for: Reference set of Mycobacterium tuberculosis clinical strains: A tool for research and product development
Source: PLoS One. 2019 Mar 25;14(3):e0214088. doi: 10.1371/journal.pone.0214088 (PMC6433267; doi:10.1371/journal.pone.0214088)
Supplement: S2 Fig — (DOCX) [file pone.0214088.s004.docx]

S2 Figure

| 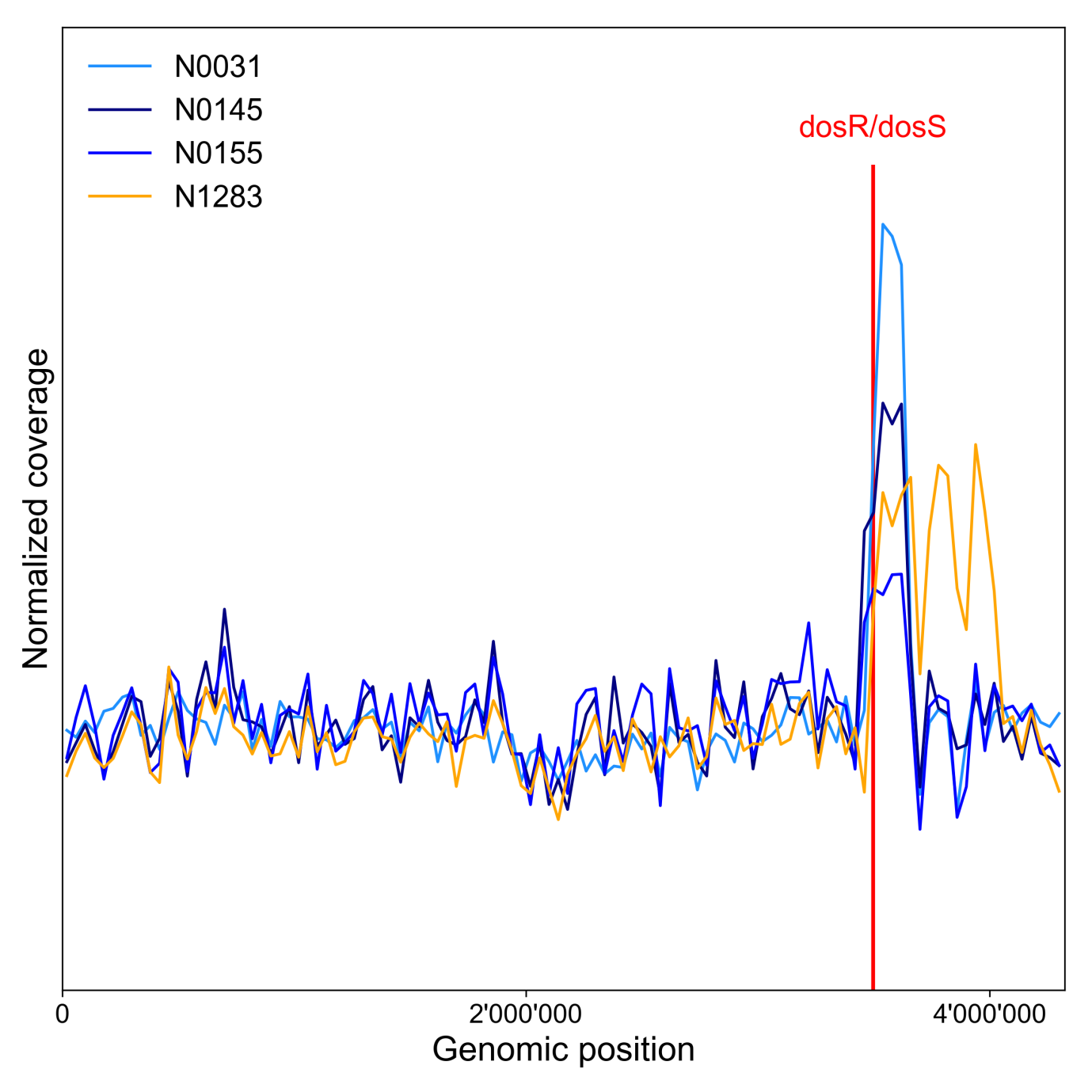 |
| --- |
| Genome duplications affecting *dosR*/*dosS* overlap across strains. We overlaid median coverage depth traces spanning 40’000 base pair bins (red in S1 Figure) for strains carrying the large genomic duplication affecting *dosR/dosS* whose location is highlighted by the red vertical line to illustrate that they impact overlapping regions of the genome. |
